# A narrative review of autophagy in migraine

**DOI:** 10.3389/fnins.2025.1500189

**Published:** 2025-02-14

**Authors:** Yanan Huang, Hongyan Li, Qijun Yu, Yonghui Pan

**Affiliations:** Department of Neurology, the First Affiliated Hospital of Harbin Medical University, Harbin, China

**Keywords:** autophagy, migraine, PI3K/Akt pathway, P2X7R, central sensitization

## Abstract

**Background and objective:**

Autophagy is a natural process regulated by autophagy-related genes in eukaryotic cells that involves the degradation of cytoplasmic proteins and old or damaged organelles via the lysosomal pathway to help maintain cell homeostasis. Previous studies have suggested a potential association between autophagy and migraine, while the underlying mechanisms remain unclear. This review seeks to evaluate the possible involvement of autophagy in the pathophysiology of migraine, aiming to clarify its role and implications for future research and therapeutic strategies.

**Methods:**

A search in PubMed was conducted for English-language articles until December 5, 2024. Key terms of “autophagy,” “migraine,” “microglia,” “neurogenic inflammation,” “central sensitization,” “mitophagy” and “neuropathic pain” in different combinations.

**Results:**

In the context of migraine, the activation of the phosphoinositide 3-kinase (PI3K)/protein kinase B (PKB/Akt) signaling pathway exerts a direct influence on the mammalian target of rapamycin (mTOR), leading to a reduction in autophagy levels. Moreover, the stimulation of purinergic ligand-gated ion channel type 7 receptor (P2X7R) in microglia can hinder autophagy by interfering with the fusion of autophagosomes and lysosomes, which impedes the degradation of substrates within the autophagolysosome. Increased levels of calcitonin gene-related peptide (CGRP) may also modulate autophagy through the Akt/mTOR or protein kinase A (PKA)/mTOR signaling pathways. Additionally, research indicates that mitophagy may be partially impaired in individuals suffering from migraine. Furthermore, autophagy could contribute to the dysregulation of synaptic plasticity by influencing the processes of long-term potentiation (LTP) and long-term depression (LTD), both of which are associated with central sensitization in chronic migraine.

**Conclusion:**

These findings suggest that autophagy may play an important role in the pathophysiology of migraine, particularly in its development and central sensitization. Research on autophagy modulators related to migraine will provide valuable insights for treatment strategies.

## 1 Introduction

Migraine is a common and recurrent neurovascular disorder characterized by unilateral, throbbing, and moderate-to-severe intensity headache, often accompanied by nausea, vomiting, photophobia, and/or phonophobia (Headache Classification Committee of the International Headache Society (IHS), 2018). It is believed to be influenced by both genetic and environmental factors (Loder and Renthal, 2019). The annual prevalence of migraine worldwide is approximately 15%, southeast Asian countries have the highest incidence, ranging from 25 to 35% (Ashina et al., 2021). The global years lived with disability (YLDs) caused by headaches amounted to 46.6 million in 2019 with migraine accounting for 88.2% (Steiner et al., 2020). According to the Global Burden of Disease Study in 2021, migraine ranked as the third highest age-standardized disability-adjusted life years (DALYs) disorder affecting the nervous system; DALYs is the sum of years of life lost (YLLs) and YLDs (GBD 2021 Nervous System Disorders Collaborators, 2024). The World Health Organization (WHO) considers severe migraine together with dementia, quadriplegia, and acute psychosis as the four most serious functional disorders (Blumenfeld et al., 2011). Currently, migraine is still considered an incurable condition, and patients with recurrent migraine may experience psychological issues such as anxiety and depression (Kocakaya et al., 2023).

Autophagy is a highly conserved catabolic process induced by various stresses, including hypoxia, nutrient deprivation, inflammation, and other cytotoxic insults, that prevents cellular damage and promotes cell survival (Dikic and Elazar, 2018). Moderate autophagy is essential for maintaining cellular homeostasis and promoting cell survival. However, disturbances in the level or regulation of autophagy can result in excessive or insufficient autophagy, thereby disrupting the normal turnover of cellular components and contributing to various disease states. Autophagy is implicated in numerous human diseases, such as cancer, inflammatory diseases, and autoimmune diseases (Mizushima and Levine, 2020).

Recent studies have found that autophagy also plays an important role in the nervous system (Fleming and Rubinsztein, 2020; Eshraghi et al., 2021; Shen et al., 2015):

- Core autophagy genes have been confirmed to be expressed in the central nervous system.
- The formation and regulation of synapses, as well as synaptic plasticity, are closely linked to autophagy.
- Autophagy mediators coordinate microglia phagocytosis through various mechanisms.

Among neurological diseases, autophagy is closely associated with neurodegenerative diseases such as Alzheimer’s disease (AD), Parkinson’s disease (PD), Huntington’s disease (HD), and amyotrophic lateral sclerosis (ALS; Meng et al., 2019). Additionally, autophagy is also thought to be involved in the onset and progression of ischemic stroke (Peng et al., 2022; Su et al., 2022; Xiaoqing et al., 2023).

In the context of chronic pain, autophagy dysfunction is a key factor underlying neuropathic pain, and the upregulation of autophagy has been shown to alleviate neuropathic pain (Meng et al., 2019; Liu et al., 2019). Several studies have identified autophagy dysfunction in chronic migraine models, suggesting that the activation of autophagy may exert a preventive effect on the chronification of migraine (Jiang et al., 2021; Niu et al., 2021). In this narrative review, we first provide a basic introduction to autophagy and examine the potential connection between autophagy and migraine, focusing on inflammatory pathways, glial cell receptors, and CGRP, among other factors. Subsequently, we summarize the roles of various means of autophagy modulation in migraine and neuropathic pain. This review aims to enhance the understanding of the pathological mechanisms underlying migraine from the perspective of autophagy and to explore the clinical translational potential of related autophagy interventions.

## 2 Methods

We retrieved relevant literature until December 5, 2024, from the database of PubMed. Key words of “autophagy,” “migraine,” “microglia,” “neurogenic inflammation,” “inflammatory pain,” “central sensitization,” “mitophagy” and “neuropathic pain” in different combinations. The summary of the search strategy is provided in Table 1.

**Table 1:** The search strategy summary.

Items	Specification
Date of search	December 5, 2024
Databases	PubMed
Search terms used	“autophagy,” “migraine,” “microglia,” “neurogenic inflammation,” “inflammatory pain,” “central sensitization,” “mitophagy” and “neuropathic pain”
	Search term example: (autophagy) AND (migraine)
Timeframe	Until December 5, 2024
Inclusion and exclusion criteria	We selected articles focused on autophagy studies primarily related to migraine, including those associated with chronic pain, such as inflammatory pain and neuropathic pain, while excluding other specific pain types (e.g., cancer pain, chronic pain following surgery, etc.). Both clinical studies and basic research are included in this review. Articles published in languages other than English were excluded.
Selection process	Two independent reviewers (YH and HL) excluded articles after reading the article titles and abstracts and assessed the full text to exclude articles that did not focus on migraine as a primary research topic. The reviewers resolved disagreements by negotiation or by consulting reviewer YP for comments.

## 3 Autophagy

### 3.1 The history of autophagy

Clark first reported the existence of numerous dense structures with membranous formations in the kidney tissue cells of newborn mice in 1957, which contained small canalicular structures, dense lamellar inclusions, and altered mitochondria (Jr, 1957). Later in 1962, Porter et al. discovered lysosomes in rat liver cells increased following glucagon stimulation. They also observed that some lysosomes containing components from other organelles, such as mitochondria, moved to the center of the cell. However, they considered this phenomenon to be merely a part of the lysosome formation process and did not recognize lysosomes as organelles akin to mitochondria, believing instead that the observed hydrolases were produced by microbodies (Ashford and Porter, 1962). Referring to the findings of Hruban et al., it was noted that a continuous process, starting from cytoplasmic fusion to the creation of lysosome can be induced in cell damage, cell differentiation, organelle disposal, and the reuse of cellular components (Hruban et al., 1963). In 1963, De Duve suggested that this process, which involves delivering intracellular cytoplasm and organelles to the lysosome, could be termed “autophagy.” Additionally, De Duve proposed that lysosome plays a significant role in glucagon-induced liver cell degradation (De Duve, 1963). Although researchers had begun to acknowledge the role of autophagy and lysosome in cells during this period, it was not until Ohsumi reported that nutrient starvation in yeast cells led to widespread autophagic degradation of cytoplasmic components, and that various yeast mutants could be utilized to identify and study the genes involved in the autophagy process, that autophagy garnered significant attention from biologists (Takeshige et al., 1992).

### 3.2 Classification and process of autophagy

**Figure 1:**
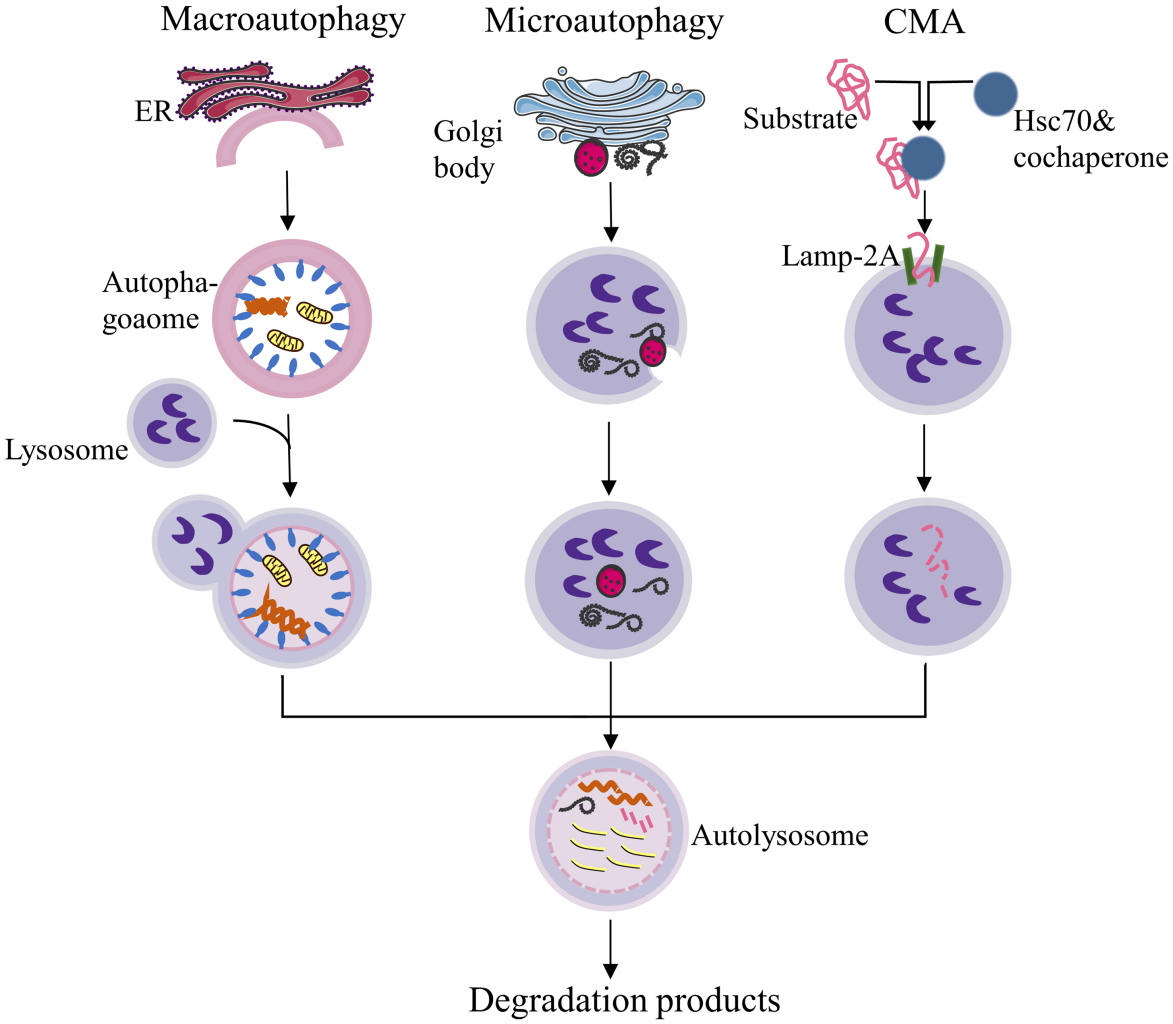
Classification of autophagy. CMA: Chaperone-mediated autophagy. ER: Endoplasmic reticulum. This figure was redrawn based on a conceptual design inspired by publicly available content, as seen on non-academic platforms (Zhihu, https://zhuanlan.zhihu.com/p/93988622).

Current research on autophagy encompasses various areas of pathology, and the role of autophagy homeostasis in human diseases is gradually becoming clearer. Autophagy occurs when certain cytoplasmic components, organelles, and proteins within cells that need to be degraded are enveloped by membranes to form autophagosomes. These autophagosomes then fuse with lysosomes to create an autophagic-lysosomal system, which decomposes inclusion bodies to maintain the stability of the intracellular environment and facilitate the renewal of organelles (Cao et al., 2021). Autophagy can be broadly categorized into three types based on the different mechanisms of transporting intracellular substrates to lysosomes (Mizushima and Komatsu, 2011). (Figure 1):

- Macroautophagy: This process involves the formation of autophagosomes with a double-membrane structure that encapsulates intracellular substances and ultimately fuses with lysosomes.
- Microautophagy: This mechanism directly engulfs specific organelles through deformation of the lysosomal or vacuolar surface.
- Chaperone-mediated autophagy (CMA): In this process, proteins with KEFRQ-like motifs are transported to lysosomes via the lysosomal-associated membrane protein 2A (LAMP-2A) transporter with the assistance of heat shock protein 70 (HSP70) chaperones.

The term “autophagy” typically refers to “macroautophagy,” which is characterized by the formation of autophagosomes. Additionally, there is a cellular process that requires the direct binding of specific receptor proteins to the autophagy protein light chain 3 (LC3) or autophagy-related proteins (Atg) 8. This binding facilitates the transport of intracellular protein aggregates or damaged organelles, such as mitochondria, to lysosomes or vacuoles for degradation (Liu et al., 2014). This mode of autophagy is named as selective autophagy. Generally, the morphology of selective autophagy is similar to that of non-selective autophagy. The primary distinction is that selective autophagy degrades specific substrates. Currently, recognized forms of selective autophagy include mitophagy, lysophagy, reticulophagy, nucleophagy, among others (Yao et al., 2021).

**Figure 2:**
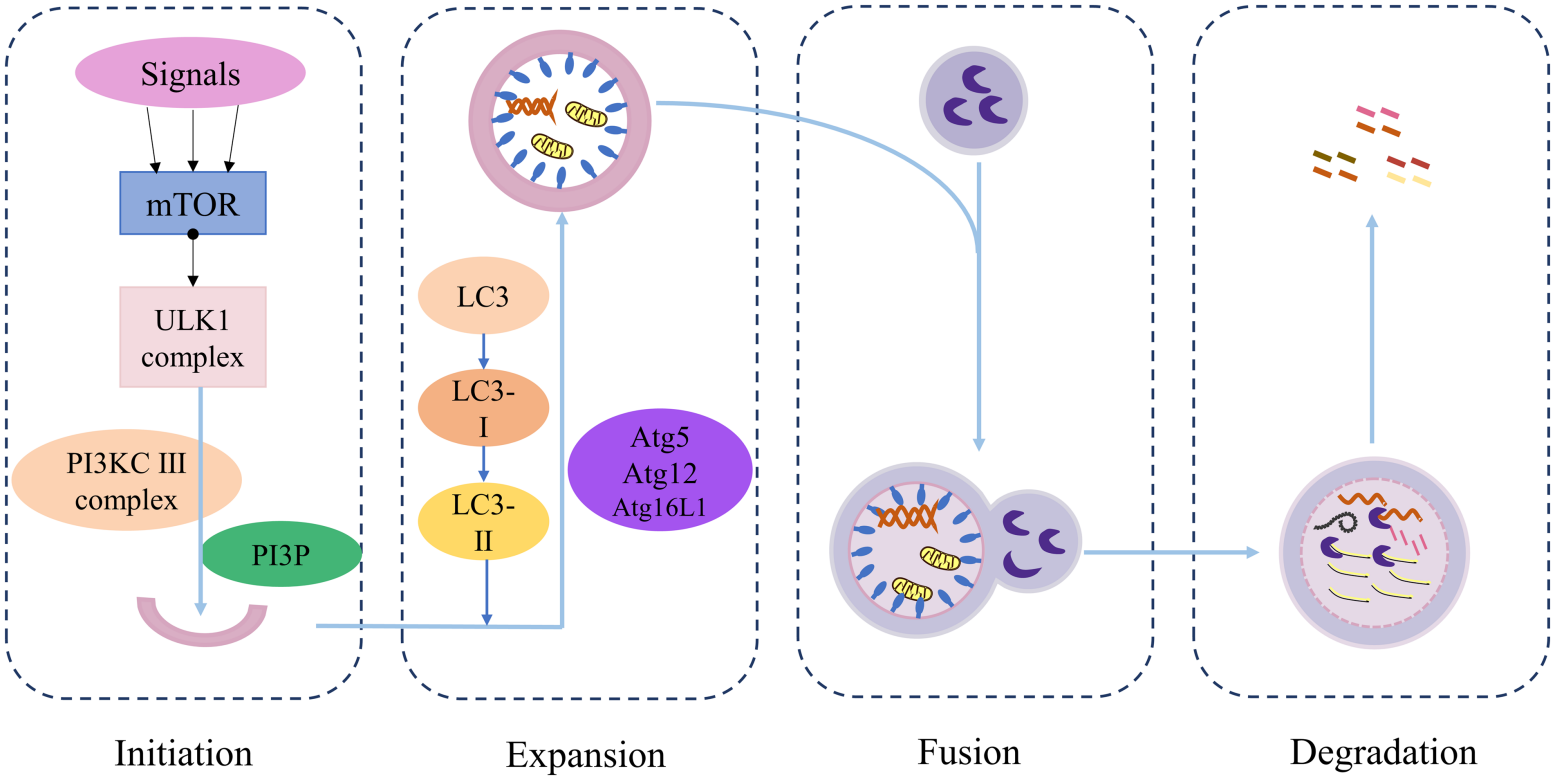
Overview of the autophagy process.

It was initially believed that the primary function of autophagy-related genes was to coordinate and mediate the formation of bilayer structures that transport cytoplasmic contents to lysosomes for degradation, a process that is conserved across all eukaryotes. However, more recent studies have revealed that, in addition to autophagy, autophagy-related genes are increasingly implicated in cell death pathways, cell cycle regulation, and innate immune signal transduction, as well as phagocytosis, secretion, exocytosis, and membrane transport (Levine and Kroemer, 2019). Autophagy mainly consists of four stages: initiation, extension, fusion, and degradation (Figure 2): (1) Initiation: Under conditions such as starvation, injury, stress, etc., autophagy is activated. The induction signal targets the mTOR to activate the Unc-51-like kinase 1 (ULK1) complex and the class III PI3K (PI3KC3) complex, triggering the emergence of autophagy. (2) Extension: The AtG5-ATG12-ATG16L complex, which is associated with LC3 proteins ubiquitin-like conjugation system, collectively promotes the elongation of the autophagosome membrane. (3) Fusion: The autophagosome membrane continually captures proteins, organelles, and other substances that need to be degraded or removed in the process of extension, forming closed spherical structures called autophagosome. Mature autophagosome then fuses with a lysosome to form an autophagolysosome. (4) Degradation: Acid hydrolases within the lysosome degrade the contents of the autophagolysosome, and the resulting degradation products are transported to the cytoplasm for reuse by the cell (Dikic and Elazar, 2018).

### 3.3 Signal pathways of autophagy

**Figure 3:**
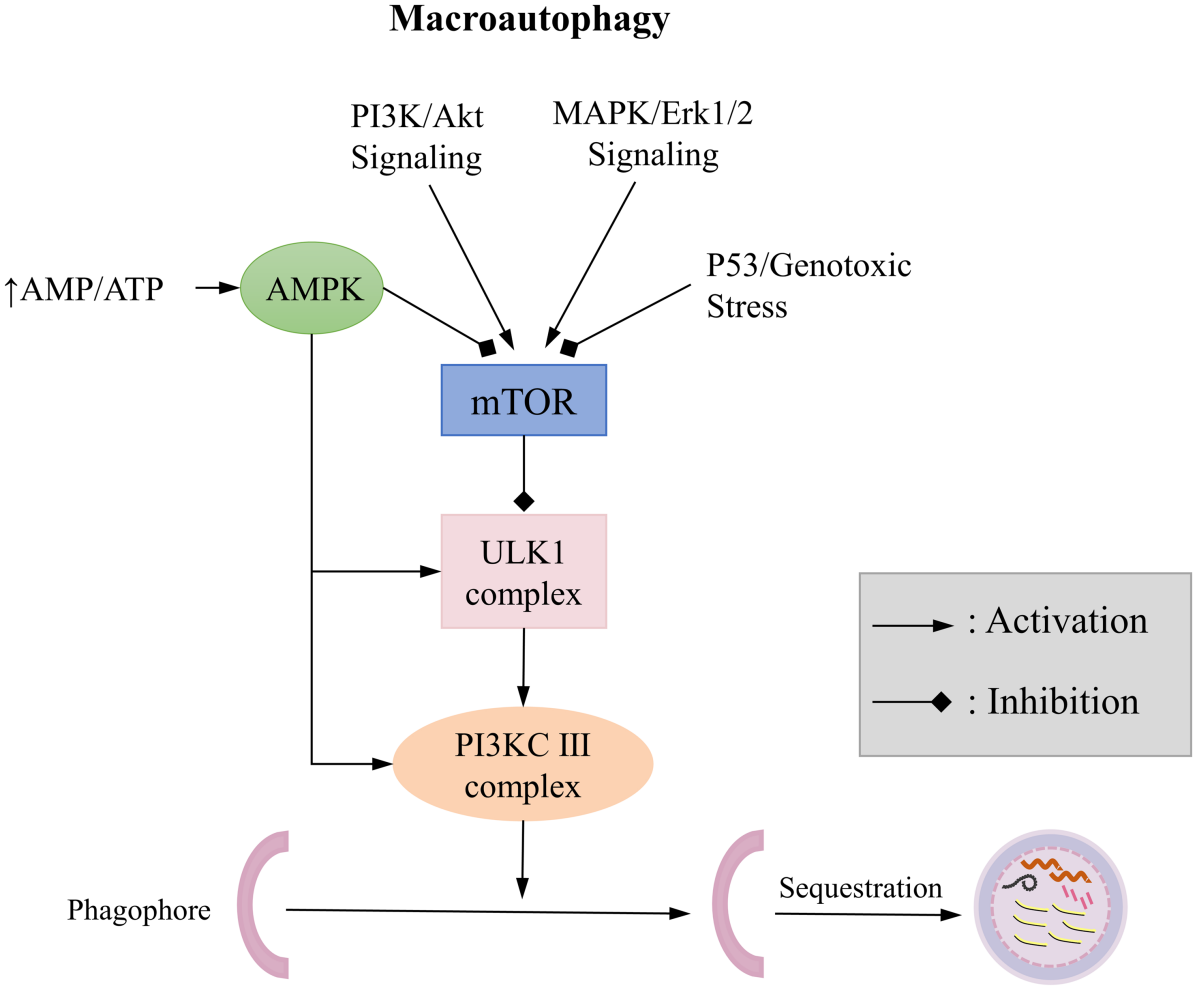
Typical Signaling Pathways of Macroautophagy.

It is well known that autophagy is a response to various environmental stress, such as nutrient deficiency, growth factor deficiency, and hypoxia. The induction of autophagy helps eliminate the damage caused by these stress, allowing the cell to return to normal levels once the stress is alleviated. Understanding the regulation of autophagy is essential for comprehending its induction under various stress conditions and its impact on cellular life processes. Many signaling pathways are involved in the regulation of autophagy, which are described detaily in the book edited by Qin, mainly focused on targets of rapamycin (TOR; Qin, 2019). Here, we refer to the Cell Signaling Technology website^1^ for a brief overview of autophagy-related signaling pathways (Figure 3): The mTOR kinase is a critical regulator of autophagy; activated mTOR (via Akt and mitogen-activated protein kinase (MAPK) signaling) inhibits autophagy, while negative regulation of mTOR (through Adenosine 5′-monophosphate (AMP)-activated protein kinase (AMPK) and p53 signaling) promotes autophagy.

## 4 Autophagy and migraine

Numerous studies have explored the relationship between autophagy and chronic pain, such as neuropathic pain, inflammatory pain, and fibromyalgia (Liu et al., 2019; Manivasakam et al., 2023; Mokhemer et al., 2023), however, its correlation with migraine is still in its stages. The trigeminal vascular theory is the classic explanation for migraine, positing that when the unmyelinated fibers of trigeminal nerve in the dura mater and dural vessels are stimulated by harmful stimuli, the trigeminal vascular system transmits this injurious information to the central regions of the brain. This process is often accompanied by the release of various neurotransmitters, including CGRP, pituitary adenylate cyclase-activating polypeptide (PACAP 38), glutamate, and nitric oxide (NO), which lead to peridural vasodilation, plasma protein extravasation, and mast cell degranulation, thereby triggering a series of neurogenic inflammatory responses that result in migraine (Dodick, 2018).

The relationship between autophagy and neurogenic inflammatory response has been previously established in other neurological disorders (Cheng et al., 2023). Therefore, it is worthwhile to investigate whether a similar relationship exists between autophagy and migraine, given that migraine also involves neurogenic inflammatory response. Upon reviewing the literature, we identified potential interconnections between autophagy and various migraine-associated inflammatory pathways, glial cells, factors, or receptors.

### 4.1 Autophagy and the PI3K/Akt pathway in migraine

The classic inflammatory signaling pathway PI3K/Akt has been confirmed to be activated in the pathogenesis of migraine and is also involved in various inflammatory responses. A recent clinical study involving 226 migraine patients and 452 non-migraine control subjects elucidated the role of PI3K/Akt signaling pathway-related genes in migraine, identifying novel associations of *RASGRP2-rs2230414, PIK3R1-rs3730089, CACNA1H-rs61734410, PRKCA-rs2286674,* and *G6PC2-rs3732033* with migraine susceptibility (Wang et al., 2024). The PI3K/Akt pathway regulates neural signaling and promotes inflammation, contributing to the development of migraine. In another study investigating differential circular RNAs (circRNAs) in migraine patients, significant enrichment of the PI3K/Akt signaling pathway was also observed (Lin et al., 2020). Through transcriptome sequencing of the hypothalamus in chronic migraine mice and control mice, Gong et al. found that differentially expressed genes were predominantly enriched in the PI3K/AKT/mTOR pathway (Gong et al., 2024). Furthermore, Liu et al. (2017) reported that in a rat model of migraine established by intraperitoneal injection of nitroglycerin (NTG), the expression of the PI3K gene significantly increased within 6–24 h post-administration, and the expression of p-Akt (S473) in the migraine model group was markedly higher than that in the control group. Additionally, Mao et al. (2022) found that San Pian decoction can alleviate NTG-induced migraine by modulating the PI3K/Akt signaling pathway. Wu et al. (2022) based on network pharmacology and molecular docking concluded that the effective drug components of the saposhnikovia divaricata-Angelica dahurica herb pair can treat migraine by acting on PI3K/Akt target. The above studies suggest that the PI3K/Akt signaling pathway plays a significant role in the pathogenesis of migraine, and regulating this pathway may provide a promising therapeutic approach for migraine treatment.

Meanwhile, the PI3K/Akt pathway is also one of the autophagy-regulated pathways, with its activation of the downstream effector mTOR directly involved in the regulation of autophagic response (Wang et al., 2019). PI3K possesses serine/threonine (Ser/Thr) kinase activity and phosphatidylinositol kinase activity. Based on structure, function, substrates, and products, PI3K is classified into three main groups (Thibault et al., 2023): Class I PI3K activates the Akt/mTOR pathway primarily by generating phosphatidylinositol-3,4,5-trisphosphate (PIP3), which regulates various functions such as proliferation, migration, differentiation, and metabolism. Class II PI3K, which has been less extensively studied, produces lipid products including PI3P and phosphatidylinositol (4,5) bisphosphate (PIP2), predominantly found in intracellular vesicles, and participates in vesicle transport, membrane transport, insulin signaling, and receptor internalization. Vps34 is the sole member of Class III PI3K, phosphorylates phosphatidylinositol (PI) to PI3P, and is involved in the regulation of autophagy, endocytosis, and phagocytosis. Activation of the PI3K/Akt/mTOR pathway primarily negatively regulates autophagy. For instance, Akt-mediated inhibition of FoxO3 activity hinders the initiation of autophagy by decreasing the expression of FoxO1 protein, which binds and activates autophagy proteins such as ATG7. mTOR inhibits the formation of the ULK I complex by phosphorylating ULK1 and Atg13, as well as inhibiting the formation of the PI3KC III complex by phosphorylating Atg14, AMBRA1, and NRBF2. Additionally, mTOR inhibits LC3 acetylation and WIP2 ubiquitinylation catalyzed through the phosphorylation of P300 and WIP2 (Cheng, 2019; Dossou and Basu, 2019). The anti-oncogene PTEN plays a regulatory role in nociceptive hypersensitivity and injurious sensation, inhibiting the activation of the PI3K/Akt signaling pathway to promote cell autophagy (Pulido, 2018).

In summary, it is evident that autophagy inhibition mediated by the activated PI3K/Akt signaling pathway may contribute to the developmental process of migraine. However, further studies are necessary to clarify the specific pathways and modalities that play key roles in the multiple pathways involved in this signaling cascade.

### 4.2 Autophagy and microglia in migraine

Microglia, the primary immune cells of the central nervous system, are responsible for releasing variety of neurotransmitters and pro-inflammatory factors upon activation. They interact with neurons and influence neuronal excitability, playing a crucial role in the chronicity of migraine (Sudershan et al., 2023). The activation of microglia depends on various receptors and signaling molecules, including purinergic receptors, Toll-like receptor (TLR), chemokine receptors, MAPK, and transcription factors (Chen et al., 2018). Fried et al. (2018) conducted a study in which they simulated recurrent episodes of migraine by repeatedly stimulating the dura mater with inflammatory soup (IS). They observed microglial activation in the trigeminal nucleus caudalis (TNC) and an increase in blood–brain barrier permeability during the chronic phase of migraine. Additionally, they found that inhibiting microglial function with minocycline prior to the infusion of IS significantly improved pain behavior in the model rats. Liu et al. (2018) discovered that the expression of alpha 7 nicotinic acetylcholine receptor (α7nAChR) was reduced in a chronic migraine rat. They also found that the activated α7nAChR could increase the mechanical pain threshold and reduce the pain manifestation of the model rats by down-regulating microglial and astrocyte activation. In another study, Won and Kraig (2021) administered insulin-like growth factor-1 (IGF-1) intranasally in a rat model of migraine induced by intraperitoneal injection of NTG. They observed that IGF-1 reduced the susceptibility to cortical spreading depression (CSD) by blocking oxidative stress and neuroinflammation in microglia. Furthermore, IGF-1 was found to inhibit activation of the trigeminal nervous system, suppress CGRP expression, and reduce susceptibility to CSD by mitigating oxidative stress and neuroinflammation in microglia. These studies collectively suggest that microglia play a significant role in migraine through various mechanisms.

Several studies have indicated that microglial autophagy may play a crucial role in central nervous system (CNS) diseases, with a primary focus on neurodegenerative diseases such as AD and PD. These findings suggest that microglial cells can maintain neuronal function and promote neuroprotection by removing amyloid-beta (Aβ) and extracellular alpha-synuclein (α-syn) from neurons through the process of autophagy (Wang et al., 2023). Jiang et al. (2021) found that autophagy dysfunction was also present in chronic migraine. In mice subjected to repeated NTG administration, the ratio of LC3-II/LC3-I in the TNC was significantly increased, along with elevated expression of p62, suggesting impaired autophagic flux, specifically, autophagy initiation was normal but downstream processes were blocked. Additionally, the expression of P2X7R was increased and predominantly co-localized with microglia in the TNC of this model. The application of a selective P2X7R antagonist activated autophagic flux levels, ameliorated basal nociception and acute nociceptive sensitization, reduced the expression of CGRP and c-fos in the TNC, and inhibited the activation of microglial cells and nucleotide-binding oligomerization domain-like receptor protein 3 (NLRP3) inflammasome. P2X7R is an ATP-gated non-selective cation channel belonging to the purinergic receptor P2X family. In microglia, P2X7R influences both intra-and extracellular clearance by modulating lysosome function, phagocytosis, and autophagy, and it promotes NLRP3 inflammasome activation through activating cathepsin B. The effects of long-term activation of microglia P2X7R on autophagy are primarily reflected in the following ways: (i) it may reduce the fusion of autophagosome and lysosome; (ii) it induces lysosomal de-acidification by increasing lysosomal pH, resulting in decreased autophagy; (iii) it directly up-regulates cellular expression of LC3-II and p62, which facilitates autophagosome formation while inhibiting the degradation of the substrate in the late-phase autophagolysosome, thus inhibiting autophagy (Campagno and Mitchell, 2021).

Therefore, by establishing the migraine-microglia P2X7R-neuroinflammation-autophagy axis, we have found that microglial autophagy dysfunction is likely to be involved in the pathogenesis of migraine. Furthermore, the direct application of the P2X7R agonist BzATP to microglia can trigger AMPK activation and high LC3II expression, as well as inhibit mTOR via the reactive oxygen species (ROS) and calcium/calmodulin-dependent protein kinase II (CaMKKII) pathways (Fabbrizio et al., 2017; Sekar et al., 2018), whether this process can regulate autophagy through the classical AMPK/mTOR pathway needs further investigation.

### 4.3 Autophagy and CGRP

The critical role of CGRP in migraine has been acknowledged by numerous researchers, and the prophylactic treatment of migraine with CGRP receptor antagonists has emerged as a prominent area of research in recent years. Multiple clinical studies have demonstrated that peripheral blood CGRP levels are elevated acute attacks of acute migraine and chronic migraine, and in the absence of diagnostic evidence, CGRP appears to serve as a biomarker to assist in the diagnosis of migraine (Cernuda-Morollón et al., 2013; Cho and Chu, 2023; Ramón et al., 2017). CGRP receptor antagonists (Ubrogepant, Atogepant; Ailani et al., 2021; Lipton et al., 2019), CGRP-targeted monoclonal antibodies (Eptinezumab, Fremanezumab, and Galcanezumab; Ashina et al., 2022; Silberstein et al., 2017; Zhou et al., 2023), and monoclonal antibodies against the CGRP receptor (Erenumab; Goadsby et al., 2017) are effective in relieving and prophylactically treating migraine. CGRP is a neuropeptide composed of 37 amino acids, primarily secreted by C and Aδ sensory fibres (Xiong et al., 2023). There are two subtypes of CGRP: αCGRP and βCGRP. αCGRP is generally regarded as the predominant form in the central and peripheral nervous system, while βCGRP is mainly located in the enteric nervous system (Hendrikse et al., 2019; Mulderry et al., 1985). In migraine, in addition to its potent vasodilatory effects, CGRP is also involved in the process of central sensitization, where central CGRP can activate neuroglia to maintain inflammatory responses and neural hypersensitivity through a positive feedback mechanism. Furthermore, it can enhance the central glutamate signaling pathway, subsequently increasing synaptic plasticity and decreasing pain threshold sensitization (Russo and Hay, 2023).

The interaction between CGRP and autophagy has been found in studies involving other diseases. For instance, in astrocytes with neuropathic pain, CGRP acts on its receptor and leads to H3K9 acetylation (H3K9ac), which is mainly associated with the expression of genes related to proliferation, autophagy, and inflammation (Sun et al., 2021). Tian et al. (2020) found that exogenous administration of CGRP attenuated neuronal apoptosis and autophagy in cortical tissues at the site of injury after traumatic brain injury (TBI), at least in part through the regulation of the Akt/mTOR signaling pathway. Schavinski et al. (2021) reported that CGRP strongly inhibited autophagy in the hearts of mice both in the basal state and during fasting following *in vivo* injection, possibly through activation of the PKA/mTORC1 signaling pathway, regardless of Akt. Although the above studies show that there is indeed an association between autophagy and CGRP, certain aspects warrant careful consideration. For instance, in the study by Schavinski (Schavinski et al., 2021), it was reported that CGRP decreased p-Akt, whereas in the experiments by Tian et al. (2020), p-Akt/Akt levels were elevated after the administration of CGRP, even though both studies ultimately demonstrated that CGRP suppressed the expression of autophagy-associated proteins. The CGRP receptor is a heterodimer consisting of the calcitonin receptor-like receptor (CLR) and receptor activity modifying protein 1 (RAMP1), in addition to another protein — receptor component protein (RCP) — that is essential for the function of CGRP receptors and which confers specific pharmacological sensitivity to CGRP (Prado et al., 2002). RCP is coupled to Gαs and activates adenylate cyclase (AC) causing an increase in intracellular cyclic adenosine monophosphate (cAMP) levels, which in turn activates PKA, leading to the phosphorylation of a variety of downstream targets, including potassium-sensitive ATP (KATP) channels, extracellular signal-regulated kinases (ERKs), transcription factors (eg, cAMP response element binding protein, CREB), and phosphorylated nitric oxide synthase (NOS; Russell et al., 2014).

Among these, autophagy could be directly influenced by activating the Erk/mTOR signaling pathway. Whether these studies can serve as a reference for the interactions between CGRP and autophagy in the context of migraine deserves further verification.

### 4.4 Autophagy and central sensitization of chronic migraine

Chronic migraine affects 1–2% of the global population, with approximately 2.5% of patients with episodic migraine progressing to chronic migraine each year (Burch et al., 2019). Chronic migraine was initially classified only as a complication of migraine in the International Classification of Headache, Version 2 (ICHD-II), and was later formally recognized as a stand-alone diagnosis alongside migraine with aura and migraine without aura in the ICHD-III published by the International Headache Society (Headache Classification Committee of the International Headache Society (IHS), 2018).

Central sensitization is an important pathophysiological mechanism in chronic migraine, mainly involving alterations in long-term synaptic plasticity, specifically LTP and LTD. At the molecular level, both LTP and LTD are dependent on calcium influx induced by the activation of N-methyl-D-aspartate (NMDA) receptors. During the formation of LTP, the expression and insertion of α-amino-3-hydroxy-5-methyl-4-isoxasolepropionic (AMPA) receptors in the postsynaptic membrane increases. Conversely, during LTD formation, AMPA receptors are rapidly endocytosed in the postsynaptic membrane, and some of the endocytosed AMPA receptors are recycled back to the postsynaptic membrane, while others are transported to the lysosome for degradation (Royo et al., 2022). Autophagy is thought to be involved in the LTP and LTD process at the postsynaptic membrane of CNS neurons. According to Kallergi et al’ research (Kallergi et al., 2022), autophagy is believed to play a significant role in LTP and LTD. The authors applied a low dose of NMDA to induce neuronal LTD and observed an increase in the number of LC3, accompanied by a decrease in the expression of AMPA receptor subunit GluA2 and postsynaptic density protein 95 (PSD95) in dendrites. This suggests that the induction of LTD triggers the initiation of autophagy in the dendrites of postsynaptic excitatory neurons. Autophagy subsequently degrades postsynaptic AMPA receptor subunits and postsynaptic proteins, thereby contributing to the maintenance of LTD. Brain-derived neurotrophic factor (BDNF) is an important regulator of LTP. Nikoletopoulou et al. (2017) investigated the effects of BDNF on neurons cultured *in vitro* and found that the levels of both forms of LC3 were reduced, while the expression of p62 was dose-dependently elevated. In contrast, in the brains of animals with knockout of BDNF-related genes, LC3 levels were increased, and p62 expression was reduced, suggesting that BDNF inhibits autophagic fluxes in the CNS. The administration of BDNF antibody for a 6-h incubation in neurons undergoing LTP resulted in the elimination of field excitatory postsynaptic potential (fEPSP) responses; however, fEPSP remained enhanced when both the BDNF antibody and autophagy inhibitor were applied. This suggests that the elimination of fEPSP due to BDNF deficiency is mediated by an increase in autophagy and that the inhibition of autophagy can reverse the elimination of fEPSP induced by BDNF deficiency, thereby restoring LTP.

Therefore, autophagy dysfunction may be implicated in the pathogenesis of chronic migraine by influencing the formation of LTP and LTD, leading to defects in synaptic plasticity and consequently affecting the formation of central sensitization.

## 5 Mitophagy and migraine

Mitochondria serve as the centre of energy metabolism in eukaryotic cells, providing the foundation for cell survival and the execution of normal biological functions with the primary function being the generation of adenosine triphosphate (ATP) by aerobic oxidation. Additionally, mitochondria are the site of reactive oxygen radical production and damage, play a role in the regulation of intracellular calcium homeostasis and are involved in the processes of apoptosis (Ould Amer and Hebert-Chatelain, 2020). Mitochondrial quality control (QC) encompasses mitochondrial dynamics (mitochondrial fission and fusion), mitochondrial biogenesis, and mitophagy—a series of processes that collectively regulate intracellular mitochondrial homeostasis (Kang et al., 2018). Mitochondrial autophagy is considered to be an autophagic process in which cells degrade mitochondria in a highly selective manner (Yao et al., 2021). Under various stress conditions, mitochondria can become damaged, leading to changes in membrane permeability and subsequent mitochondrial depolarization, inducing the activation of mitophagy-related proteins, and forming autophagosomes to wrap damaged mitochondria to create mitophagosomes. Mitophagosomes then fuse with lysosomes to form mature mitophagy-lysosomes, ultimately resulting in the gradual degradation of the mitochondria encapsulated within the mitophagosomes by lysosomal enzymes (Shen et al., 2023). Mitophagy generally includes PTEN-induced putative kinase 1(PINK1)/E3 ubiquitin-protein ligase parkin (Parkin)-mediated mitophagy and receptor-regulated mitophagy. PINK1 is the receptor for mitochondrial damage. When mitochondria are compromised, the mitochondrial membrane protein kinase PINK1 accumulates at the outer membrane and recruits intracytoplasmic Parkin to the mitochondrial membranes. Parkin, possesses E3 ubiquitin ligase activity, attaches ubiquitin chains to mitochondrial substrates, resulting in the ubiquitination of damaged mitochondria. Ubiquitinated mitochondria can be recognised by the receptor protein p62, which binds to the ubiquitin chain, subsequently, p62 interacts with LC3 on the membrane of autophagic vesicles, thereby facilitating mitochondrial autophagy (Harper et al., 2018).

Previous studies have suggested that migraine attacks are correlated with impaired brain energy metabolism, with abnormal mitochondrial function is a common cause of this impairment. Under specific environmental and triggering factors, oxidative stress and disrupted brain energy metabolism can not only trigger a migraine attack but may also influence the severity of migraine (Gross et al., 2019). A study employing phosphorus magnetic resonance spectroscopy (^31^P-MRS) found that patients with migraine exhibited a lower ratio of phosphocreatine to creatine, along with elevated adenosine diphosphate (ADP) concentrations, indicating reduced neuronal energy availability and mitochondrial dysfunction (Younis et al., 2017). A cross-sectional study involving 32 migraine patients with/without aura found that, with the exception of oxidised low-density lipoprotein (oxLDL) and hemoglobin A1c (HbA1c), most of the other markers of mitochondrial function—such as alpha-lipoic acid (ALA), total antioxidant capacity (TAC), peroxide (PerOx), thiols, and lactate—exhibited a significant percentage (>30%) of abnormalities in the venous blood of the examined patients (Gross et al., 2021). Furthermore, dietary interventions targeting one or more elements of the mitochondrial dysfunction/reduced energy production/oxidative stress triad have also been shown to be effective in migraine prevention (Antonio et al., 2018; Caminha et al., 2022; Magis et al., 2007). The above studies confirm the important role of mitochondrial function in the pathophysiology of migraine, with mitophagy serving as the primary mechanism for clearing the accumulation of functionally and structurally abnormal mitochondria. A recent study found that mitochondrial ultrastructure was impaired at the TNC in migraine mice with repeated dural injections of IS, characterized by swollen mitochondria, fewer mitochondrial cristae retained, and increased fragmented mitochondria. Measurement of mitophagy-related protein levels revealed that the expression of p62 in the TNC of the IS group was significantly elevated, while the level of PINK1 protein was significantly decreased, but there were no significant changes observed in the expression levels of Parkin and Beclin1, suggesting that mitophagy was partially impaired after repeated infusions of IS (Shan et al., 2023).

In summary, mitochondrial dysfunction in migraine has been confirmed so far, however, there is a lack of studies exploring the role of mitophagy flow disorders in migraine and their underlying mechanisms. Further in-depth research could enhance our understanding of mitochondrial homeostatic disorders in migraine.

## 6 Regulation of autophagy in chronic pain

Although alterations in autophagy have been confirmed to be involved in various diseases, there are currently no specific interventions targeting the regulation of autophagy that are directly applied in clinical settings. While rapamycin (RAPA), chloroquine (CQ), hydroxychloroquine (HCQ), and some others that have been approved for specific indications can exert effects through autophagy, they were not originally developed for autophagy modulation (Galluzzi et al., 2017). The clinical translational potential of autophagy regulators warrants further exploration to enhance patient benefits. Given the limited direct research on the relationship between autophagy regulators and migraines, we have summarized in Table 2 the pharmacological and non-pharmacological autophagy-regulating approaches related to chronic pain, including chronic migraine, inflammatory pain, and neuropathic pain.

**Table 2:** Autophagy modulatory means and their effects.

	Autophagy modulator	Disease/Model	Targets/Pathway of action	Effects
Pharmacological regulation	Metabolic glutamate receptor 5 (mGluR5) (Niu et al., 2021)	Chronic migraine rat model was established by repeated infusions of IS	LC3-ІІ, p62; mTOR pathway	Downregulation of mGluR5 activated autophagy by inhibiting the mTOR pathway which alleviated allodynia and central sensitization in CM rats.
	P2X7R (Jiang et al., 2021)	Chronic migraine mice model was established by repeated intraperitoneal injection of NTG	LC3-ІІ, p62	P2X7R negatively regulated the autophagic flux in CM.
	Picropodoph-yllin (PPP) (Wang et al., 2024)	Chronic migraine mice model was established by administering intraperitoneal injections of NTG every other day	p62; mTOR pathway	Targeted intervention in the IGF/IGF1R signaling pathway through PPP can alleviate pain behaviors and improve mTOR-related autophagic dysfunction.
	Huc-MSCs-derived exosomes (Hua et al., 2022)	Inflammatory pain mice induced by complete Freund’s adjuvant (CFA)	LC3-II, beclin1, p62; miR-146a-5p/TRAF6	Huc-MSCs-derived exosomes inhibited pyroptosis through promoting autophagy via miR-146a-5p/TRAF6 in CFA-induced inflammatory pain.
	3,5-dicaffeoylquinic acid (3,5-DCQA) (Park et al., 2022)	Inflammatory pain mice induced by CFA	LC3 II, p62; MCP3/JAK2/STAT3	3,5-DCQA attenuated inflammation-mediated pain hypersensitivity by enhancing autophagy through inhibition of MCP3-induced JAK2/STAT3 signaling.
	RAPA (Yuan et al., 2024)	Trigeminal neuropathic pain (TNP) rat model induced by infraorbital nerve chronic constriction injury (IoN-CCI)	LC3I, LC3II, p62	Autophagy agonists inhibited microglia activation and alleviated nociceptive behaviors.
	OTULIN (Gumby/FAM105b) (Wang et al., 2024)	TNP rat model induced by IoN-CCI	LC3II/LC3I	OTULIN significantly mitigated neuropathic pain and neuroinflammation by activating the autophagy pathway and inhibiting the activity of the NLRP3 inflammasome.
	GSK126 (selective inhibitor of EZH2) (Meng et al., 2020)	Neuropathic pain (NP) rat model induced by left brachial plexus avulsion (BPA)	LC3II, p62; mTOR-dependent signaling pathway	Pharmacologically inhibiting EZH2 (enhancer of zeste homolog 2) promoted the suppression of autophagy induced by BPA.
	Minocycline (Qiao et al., 2023)	NP rat model induced by spinal cord injury (SCI)	LC3B, Beclin-1, p62; PI3K / Akt / mTOR	Minocycline could improve neuropathic pain induced by SCI through activating autophagy and inhibiting the PI3K/Akt/mTOR pathway.
	Resolvin D2 (RvD2) (Yang et al., 2023)	NP rat model induced by SCI	LC3-ІІ, p62; NF-κB pathway	RvD2 treatment could recover the autophagy flux via suppressing NF-κB-modulated miR-155 expression.
	Aspirin-triggered Resolvin D1 (AT-RvD1) (Wang et al., 2023)	NP rat model induced by spinal nerve ligation (SNL)	LC3BII/LC3BI, Beclin1	AT-RvD1 prevented or treated NP by relieving the activation of the NLRP 3 inflammasome by inducing autophagy regulated by the Akt and ERK signaling pathways.
	Hydrogen-rich saline (Chen et al., 2019)	NP rat model induced by SNL	LC3-II, LC3-I, Beclin-1, p62	Hydrogen activated the autophagy process by increasing the LC3 and Beclin1 expression and attenuated the inflammasome activation by down-regulation of NLRP3 and ASC expression.
	Modified citrus pectin (MCP) (Ma et al., 2016)	NP rat model induced by SNL	LC3-II, p62	MCP treatment resulted in a decreased mechanical and cold hypersensitivity in SNL rats, at least in part by regulating autophagy.
	miR-195 [101] (Shi et al., 2013)	NP rat model induced by SNL	LC3-I, LC3-II, p62, ATG14; miR-195/autophagy signaling	miR-195 markedly inhibited ATG14 protein expression in spinal microglia, and suppressed autophagy by the regulation of ATG14.
	TRIM28 (Tang et al., 2024)	NP mice model induced by spared nerve injury (SNI)	LC3-II, LC3-I, p62	TRIM28 inhibited the expression of Glycogen Synthase Kinase 3 Beta (GSK3B), thereby reducing autophagy levels in microglia.
	Resveratrol (Wang et al., 2020)	NP rat model induced by SNI	p62, LC3; TREM2-autophagy axis	Resveratrol relieved neuropathic pain through suppressing microglia-mediated neuroinflammation via regulating the TREM2-autophagy axis.
	MiR-15a (Cai et al., 2020)	NP rat model induced by CCI	Beclin-1, LC-3II/LC-3I, p62; MiR-15a/AKT3	MiR-15a inhibited the expression of AKT3, thereby stimulating the autophagic process NP.
	Loganin (Cheng et al., 2022)	NP rat model induced by CCI	Beclin-1, LC3B-II, p62, LAMP2, pro-CTSD	Loganin improved impaired lysosomal function and autophagic flux to prevent CCI-induced neuropathic pain.
	miR-99b-3p (Gao et al., 2024)	NP rat model induced by CCI	LC3-ІІ, p62; miR-99b-3p/Mmp13 axis	miR-99b-3p promoted the analgesic effect by targeting Mmp13 to promote autophagy in neuropathic pain.
	circ_lrrc49 (Tang et al., 2024)	NP mice induced by CCI of the infraorbital nerve	LC3-II, p62; circ_lrrc49/Ist1/autophagy signaling axis	The knockdown of circ_lrrc49 reduced Ist1 expression, increased LC3-II, p62 levels, and autophagosomes amount.
	BoNT/A (He et al., 2023)	NP rat model induced by CCI of the sciatic nerve	LC3-II, p62	Perineural injection of BoNT/A alleviated neuropathic pain caused by CCI by promoting autophagy flow and inhibiting NLRP3 inflammasome.
	Mesenchymal stem cell-derived extracellularvesicles (MSC-Evs) (Gao et al., 2023)	NP rat model induced by CCI	PI3K / Akt / mTOR	Intrathecal injection of MSC-EVs significantly upregulated the expression of miR-99b-3p and relieved the mechanical abnormal pain caused by activated microglia in the dorsal horn of the spinal cord of CCI rats.
	Vitexin (Huang et al., 2024)	NP mice model induced by CCI	LC3-II/I, p62; S1P/S1PR1-PI3K/ Akt axis	Vitexin acted on astrocytes to attenuate S1P-induced downregulation of the PI3K/Akt signaling pathway, restore autophagy flux in astrocytes.
	Divanillyl sulfone (DS) (Shao et al., 2021)	NP mice model induced by CCI	LC3II/I, Beclin 1, p62	DS promoted rapid clearance of ROS released by dysfunctional mitochondria through mitophagy, and further suppressed the NLRP3 inflammasome activation and neuroinflammation.
	^*^Urapiocin A (Wang et al., 2023)	NP mice induced by CCI of sciatic nerve	p62, LC3, PINK1, Parkin; PINK1/Parkin	UA activated mitophagy upon spinal cord level improvement of NP in CCI mice.
	^*^Ginkgolide B (GB) (Liang et al., 2024)	NP rat model induced by CCI	PINK1, Parkin, Tom20, LC3B, Beclin1, p62; PINK1/Parkin	GB alleviated neuropathic pain by mitigating neuroinflammation through the activation of PINK1-Parkin-mediated mitophagy.
Non-pharmacological regulation	Pulsed radiofrequency (PRF) (Xu et al., 2024)	NP rat model induced by SNI	GRK2/p38 MAPK	The 45 V—PRF and 85 V—PRF increased the number of autophagic vesicles, autophagic lysosomes, and lysosomes.
	High-voltage PRF (HV-PRF) (Chen et al., 2024)	NP rat model induced by SNI	-	Dorsal root ganglion (DRG) intervention with different voltages of PRF enhanced microglial autophagy in the spinal cord dorsal horn (SCDH) in SNI rats and effectively improved NP.
	High-frequency spinal cord stimulation (HF-SCS) (Wang et al., 2022)	NP rat model induced by SNL	LAMP2, matu-CTSD, LC3 II, p62	Increasing the levels of lysosomal-associated membrane protein 2 (LAMP 2) and mature cathepsin D (matu-CTSD) to restore lysosomal function and increase autophagosomal degradation.
	Direct moxibustion (DM) (Cai et al., 2022)	Cervical spondylotic radiculopathy (CSR) model (NP model)	LC3 II, Atg 7, Bax, Bcl-2; Act A/Smads	DM promoted autophagy through ActA / Smads to reduce apoptosis and repair nerve damage and exert an analgesic effect.
	^*^Hyperbaric Oxygen (HBO) Therapy (Kun et al., 2019)	NP rat model induced by CCI	NIX, BNIP3, Drp1; CaMKKβ/AMPK	CaMKKβ/AMPK pathway might be a potential signal pathway on analgesic mechanism of hyperbaric oxygen via mitophagy.
	Electroacupuncture (EA) (Xu et al., 2022)	NP rat model induced by SNI	p62, Beclin-1, LC3II; AMPK/ mTOR	Promoting AMPK / mTOR-mediated autophagy in DRG macrophages.
	EA (Cui et al., 2022)	NP rat model induced by SNI		The analgesic impact of EA was partly related to AMPK/mTOR pathway activation and autophagy induction in microglial cells.
	Exercise (Bai et al., 2022)	NP mice model induced by CCI of the sciatic nerve	BDNF/Akt/mTOR	Exercise training might be mediated by BDNF / Akt / mTOR signaling to promote autophagy and regulate microglial polarization to promote the recovery of sciatic nerve injury in mice.

* Mitophagy.

## 7 Conclusion and perspectives

Migraine is a complex disorder not induced by a single specific mechanism. The aura phase is thought to be associated with CSD, while the headache phase is linked to the activation of the trigeminovascular system (TGVS; Dodick, 2018; Lai and Dilli, 2020). The premonitory and postdromal phases have received less research attention, but the hypothalamus may be involved in the early stages of a migraine attack (Maniyar et al., 2014). Additionally, the persistent activation of the brainstem and diencephalon following pain stimulation may be associated with the presence of the postdromal phase (Khan et al., 2021). Thus, existing animal models of migraine induced by NTG, IS, or CSD, while providing valuable insights, may not completely replicate the complex pathophysiological processes experienced by migraine patients. Future research could explore more diverse and intricate animal models to better reflect the true characteristics of migraines. Additionally, the variability of autophagy’s role across different stages of migraine remains unknown. Although studies have indicated that autophagy flow is impaired in migraine animal models, the specific mechanisms remain unclear. Future investigations should focus on elucidating the relationship between autophagy and the onset, persistence, and relief phases of migraines. This not only aids in clarifying the role of autophagy in migraines but may also provide insights for novel therapeutic targets. Furthermore, current *in vitro* studies primarily employ microglia inflammation models to investigate the connection between migraine and autophagy, however, the interactions among neurons, microglia, and astrocytes play a crucial role in the pathogenesis of migraine. Therefore, future research could consider conducting in-depth studies on the migraine—neuroglia/neuron interactions—inflammation—autophagy axis, which will enhance our understanding of the role of autophagy in migraine.

Moreover, the role of autophagy modulators in the treatment or prevention of migraines remains inadequately investigated. Among the pharmacological autophagy modulators listed in Table 2, nearly all have been validated for their therapeutic effects primarily in animal models, suggesting their potential for translational application; however, careful consideration must be given to their effects on patients and the safety of these drugs when contemplating clinical use. As the non-pharmacological autophagy modulators, HV-PRF has demonstrated significant improvements in clinical outcomes for patients with neuropathic pain (Wan et al., 2016; Wang and Song, 2022), while HF-SCS is widely utilized in the clinical management of chronic pain (Chakravarthy et al., 2018). However, both modalities necessitate a precise determination of appropriate treatment voltage and frequency to prevent patient discomfort. Additionally, DM has been practiced in China for over 4,000 years and has proven effective in alleviating neck pain (Huang et al., 2020), whereas EA is also employed as an adjunct therapy alongside medication for chronic pain patients (Mavrommatis et al., 2012); both methods require meticulous control and skilled execution by the practitioner. HBO therapy has exhibited substantial efficacy in treating complex regional pain syndrome (CRPS), chronic painful wounds, and fibromyalgia syndrome (FMS), typically demonstrating favorable tolerance and safety (Hájek et al., 2024; Pathault et al., 2024; Boussi-Gross et al., 2024). These autophagy-related interventions have yielded notable clinical benefits, and further research into the potential applications of autophagy modulators in the context of migraine will provide valuable insights for migraine management.

In summary, autophagy could be a significant factor in the development and central sensitization of migraine, particularly in relation to neurogenic inflammation. Further research on the relationship between autophagy and chronic migraine could enhance our understanding of the disease’s progression and contribute to the development of more effective treatment strategies.

## Footnotes

^1^ https://www.cellsignal.cn/pathways/autophagy-signaling-pathway
